# Epidemiological analysis of *Leishmania tropica* strains and giemsa-stained smears from Syrian and Turkish leishmaniasis patients using multilocus microsatellite typing (MLMT)

**DOI:** 10.1371/journal.pntd.0005538

**Published:** 2017-04-12

**Authors:** Mehmet Karakuş, Abed Nasereddin, Hüseyin Onay, Emin Karaca, Ahmet Özkeklikçi, Charles L. Jaffe, Katrin Kuhls, Ahmet Özbilgin, Hatice Ertabaklar, Samiye Demir, Yusuf Özbel, Seray Töz

**Affiliations:** 1 Ege University, Faculty of Medicine, Department of Parasitology, Izmir, Turkey; 2 Department of Microbiology and Molecular Genetics, IMRIC, Hebrew University - Hadassah Medical Center, Jerusalem, Israel; 3 The Core Research Facility, The Faculty of Medicine, Hebrew University of Jerusalem, Jerusalem, Israel; 4 Ege University, Faculty of Medicine, Department of Medical Genetics, Izmir, Turkey; 5 Dr. Ersin Arslan State Hospital, Microbiology Department, Gaziantep, Turkey; 6 Technical University of Applied Sciences Wildau, Division of Molecular Biotechnology and Functional Genetics, Wildau, Germany; 7 Celal Bayar University, Faculty of Medicine, Department of Parasitology, Manisa, Turkey; 8 Adnan Menderes University, Faculty of Medicine, Department of Parasitology, Aydın, Turkey; 9 Ege University, Faculty of Science, Department of Biology, Izmir, Turkey; Istituto Superiore di Sanità, ITALY

## Abstract

Turkey is located in an important geographical location, in terms of the epidemiology of vector-borne diseases, linking Asia and Europe. Cutaneous leishmaniasis (CL) is one of the endemic diseases in a Turkey and according to the Ministry Health of Turkey, 45% of CL patients originate from Şanlıurfa province located in southeastern Turkey. Herein, the epidemiological status of CL, caused by *L*. *tropica*, in Turkey was examined using multilocus microsatellite typing (MLMT) of strains obtained from Turkish and Syrian patients. A total of 38 cryopreserved strains and 20 Giemsa-stained smears were included in the present study. MLMT was performed using 12 highly specific microsatellite markers. Delta K (ΔK) calculation and Bayesian statistics were used to determine the population structure. Three main populations (POP A, B and C) were identified and further examination revealed the presence of three subpopulations for POP B and C. Combined analysis was performed using the data of previously typed *L*. *tropica* strains and Mediterranean and Şanlıurfa populations were identified. This finding suggests that the epidemiological status of *L*. *tropica* is more complicated than expected when compared to previous studies. A new population, comprised of Syrian *L*. *tropica* samples, was reported for the first time in Turkey, and the data presented here will provide new epidemiological information for further studies.

## Author summary

Turkey is one of the endemic countries for leishmaniasis, cutaneous and visceral. Cutaneous leishmaniasis caused by *Leishmania tropica* is a serious public health problem with more than two thousands of local cases each year. Moreover, with the civil war in Syria more than three million refugees were accepted to reside in different cities of Turkey. To date, several population genetic studies were performed using Turkish *L*. *tropica* isolates but all previously studied isolates were originating from the same geographical area thus population structure could not be revealed in detail for Turkey. The findings of this study suggests two new population structure for Turkey, which are consist of Syrian and Mediterranean isolates. Our findings provide important knowledge on epidemiology of *L*. *tropica* in Turkey and highlights the future role of *L*. *tropica* to Europe through the current wave of migration of infected people.

## Introduction

Leishmaniasis is a parasitic disease caused by intracellular protozoan parasite, *Leishmania* and transmitted by the bite of a certain female *Phlebotominae* sand flies. Leishmaniasis is classified as cutaneous, visceral and mucocutaneous by clinical manifestations and it is among the world’s six major tropical diseases. More than 70% of cutaneous leishmaniasis (CL) cases were reported from Afghanistan, Algeria, Colombia, Brazil, Iran, Syria, Ethiopia, North Sudan, Costa Rica and Peru [1]. In most countries cutaneous leishmaniasis is under-report, therefore it is difficult to estimate the real number of the cases. In Turkey, the first CL case was reported in 1883, and 46.003 cases recorded between 1990–2010 [2]. Cutaneous leishmaniasis is a major health problem in Turkey. According to the Turkish Ministry of Health 45% of the CL patients originate from Şanlıurfa province located in the southeastern region of Turkey. To date, four different *Leishmania* species (*L*. *tropica*, *L*. *donovani*, *L*. *infantum* and *L*. *major*) were reported in Turkey to cause CL [3,4]. Rare cases were reported that *L*. *tropica* may be the causative agent of visceral leishmaniasis (VL) in Mediterranean Basin as well as in Turkey [5].

Syrian Ministry of Health reported that incidence of CL has increased during the last 15 years and peaked in 2011 with 58.156 cases [6]. Aleppo province, Syria is a hyper-endemic area for CL with 12.000 cases reported annually [7]. Species identification of the causative agent for CL in Syria revealed that the majority of cases (85%) were caused by *L*. *tropica*, while only 15% were identified as *L*. *major* [8]. The ongoing Syrian civil war displaced more than 6.5 million people from Syria [9]. Millions of Syrian refugees have fled to neighboring countries with approximately three million refugees residing in Turkish camps alone. In Gaziantep, a city located in southeastern Turkey, reports have shown a dramatic increase in the observed number CL patients admitted to state hospitals [10]. In 2013, the number of Syrian patients admitted to hospitals due to CL peaked with 76 positive cases, as opposed to only one positive case reported in 2012 [11].

Turkish *L*. *tropica* strains obtained from CL patients in Şanlıurfa during the 1995 outbreak were analyzed using MLMT in a previous study [12]. All 27 isolates from this outbreak showed an identical genotype, and formed a microcluster together with two strains from Adana. The MLMT profile of *L*. *tropica* strains isolated from Syrian refugees in Turkey have not been determined, and it is clear from published reports that many of these refugees will only pass through Turkey on their way to Europe where potential vector sand fly species have been reported in several countries [13,14].

Previous studies using Multilocus Enzyme Electrophoresis (MLEE) analysis of Turkish *L*. *tropica* isolates, conducted by Leishmaniasis Reference Center Montpellier, France, indicated that the majority of strains belong to zymodeme MON-304. These strains were highly heterogeneous with 8 different zymodeme profiles (MON-55, MON-200, MON-303, MON-304, MON-312, MON-313, MON-314, and MON-315) noted [5]. The discriminatory power of the MLEE is limited, and not associated with geographic distribution. In addition, because large numbers of parasites are required, clinical samples cannot be used for MLEE analysis, which is important to understand the epidemiology of the disease. Another disadvantage of this method is that the enzyme panel may vary across the reference laboratories, and the obtained raw data cannot be compared directly among them.

Microsatellite or short tandem repeats (STRs) are the neutral consecutive repeats of nucleotides ranging from 1 to 6 bases in the non-coding regions of the genome. MLMT is a powerful tool for discriminating among populations and sub-populations using a battery of markers. The population genetics of different *Leishmania* species were studied previously with various numbers of specific microsatellite markers. To date, 16 different polymorphic markers were designed to reveal genetic structure and dynamics of *L*. *tropica*, but subsequently only 12 specific microsatellite markers were used to reveal genetic structure of *L*. *tropica* [15, 16, 17]. *L*. *tropica* is the most widely distributed agent of CL in the Old World, and causes many different disease pathologies including chronic and recidivans skin disease. It has been identified as the main agent responsible for CL in Turkey [2,5]. In the present study, we examined the MLMT profiles of *L*. *tropica* from cultured strains and Giemsa-stained smears obtained from Turkish and Syrian patients in different geographic regions, thus updating the epidemiological status of CL caused by this parasite in Turkey.

## Material and methods

### Ethical statement

The sample collection from patients was done according to the Ethical Committee approval (No: 2016/266) of the “Dr. Ersin Arslan State Hospital” in Gaziantep. All adult subjects provided written informed consent, and a parent or guardian of any child participant provided informed consent on their behalf.

### Geographical origin of the strains and biological samples

A total of 38 previously isolated and cryopreserved strains, and 20 Giemsa-stained smears, obtained from Turkish and Syrian patients, were included in this study. The 38 isolates were selected from our *Leishmania* Cryobank according to their geographical origins. Fifteen strains isolated during the outbreak in Şanlıurfa in 1995, 11 strains from Aydın province, two isolates (one viscerotropic) from Manisa province, five isolates from Syrian patients diagnosed in Turkey, one isolate from five different provinces (Malatya, Muş, Muğla, Niğde, Izmir) were used in the study (S1 Table). Zymodeme (MON) analysis was conducted for all autochthonous strains used in this study in Montpellier, France.

Additionally, 20 Giemsa-stained smears obtained from the lesions of CL patients were also included in the study. Twelve and eight of these samples were gathered from Syrian and Turkish patients, respectively, at the Dr. Ersin Arslan State Hospital in Gaziantep.

### DNA extractions and microsatellite typing

Cryopreserved isolates were removed from liquid nitrogen and first cultured in NNN medium for two weeks. After the mass cultivation in RPMI+10%FCS medium, promastigotes were harvested and genomic DNA was isolated using Qiagen DNeasy^®^ Blood & Tissue Kit (Qiagen, Hilden, Germany). To isolate genomic DNA from patient samples, the material was removed from the Giemsa-stained smears by washing with molecular grade water and centrifuged at 8.000 rpm for 10 minutes. After centrifugation, the supernatant was discarded and pellet used for the DNA isolation. The isolation was performed using Qiagen DNeasy^®^ Blood & Tissue Kit (Qiagen, Hilden, Germany) and the DNA was eluted in a final volume of 50 μl. Species typing of the parasite were done using the internal transcribed spacer 1 (ITS1) real-time PCR as reported previously [5]. Only samples identified as *L*. *tropica* were included in this study. Previously reported twelve highly specific microsatellite markers were used to amplify the regions containing the repeat motifs [18]. After successful amplification of the DNA, PCR products were loaded to ABI PRISM 3130XL (Applied Biosystems, USA) sequencer and fragment sizes determined using Geneious R8 [19]. The reference strain of *L*. *tropica*, MHOM/PS/2001/ISL590, was used in each run as a standard and the fragment sizes were noted for each marker. The repeat numbers were determined using the repeat motifs and numbers of the reference strain.

### Microsatellite data analysis

After the normalization step of the raw fragment data, several modelling softwares were used to determine the population structure. The data was prepared as a text file and converted into the necessary input file formats using Microsatellite Analyzer 4.05 (MSA) software. In order to determine number of the populations, Bayesian clustering method was applied using STRUCTURE V.2.3.4 [20]. The Markov Chain Monte Carlo iteration was set to 200.000 and length of burnin period to 20.000. In order to determine most appropriate number of populations, 10 iterations were applied for each K value (K1-10). Delta K (ΔK) calculation was made using an online tool, Structure Harvester [21]. The distribution of genetic variation was evaluated using Factorial Correspondence Analysis (FCA) implemented in Genetix 4.05 [22]. Genetic distances based on Chord distance were calculated using POPULATIONS 1.2 software [23]. Phylogenetic network was created using SplitsTree software [24]. Genetic distances between populations were calculated using MSA 4.05 [25]. GDA 1.1 software was used to calculate number of alleles (A), observed (H_o_), expected (H_e_) and heterozygosity and the inbreeding coefficients (F_IS_) [26].

## Results

In total 58 (38 isolates and 20 Giemsa-stained smears) *L*. *tropica* samples were used in this study and microsatellite typing were applied using 12 highly specific markers. All Giemsa-stained smears were confirmed to be *L*. *tropica* by ITS-1 real-time PCR. PCR amplification of the *L*. *tropica* samples was done and fragment analysis was performed.

### Descriptive analysis of *L*. *tropica* samples

**Fig 1:**
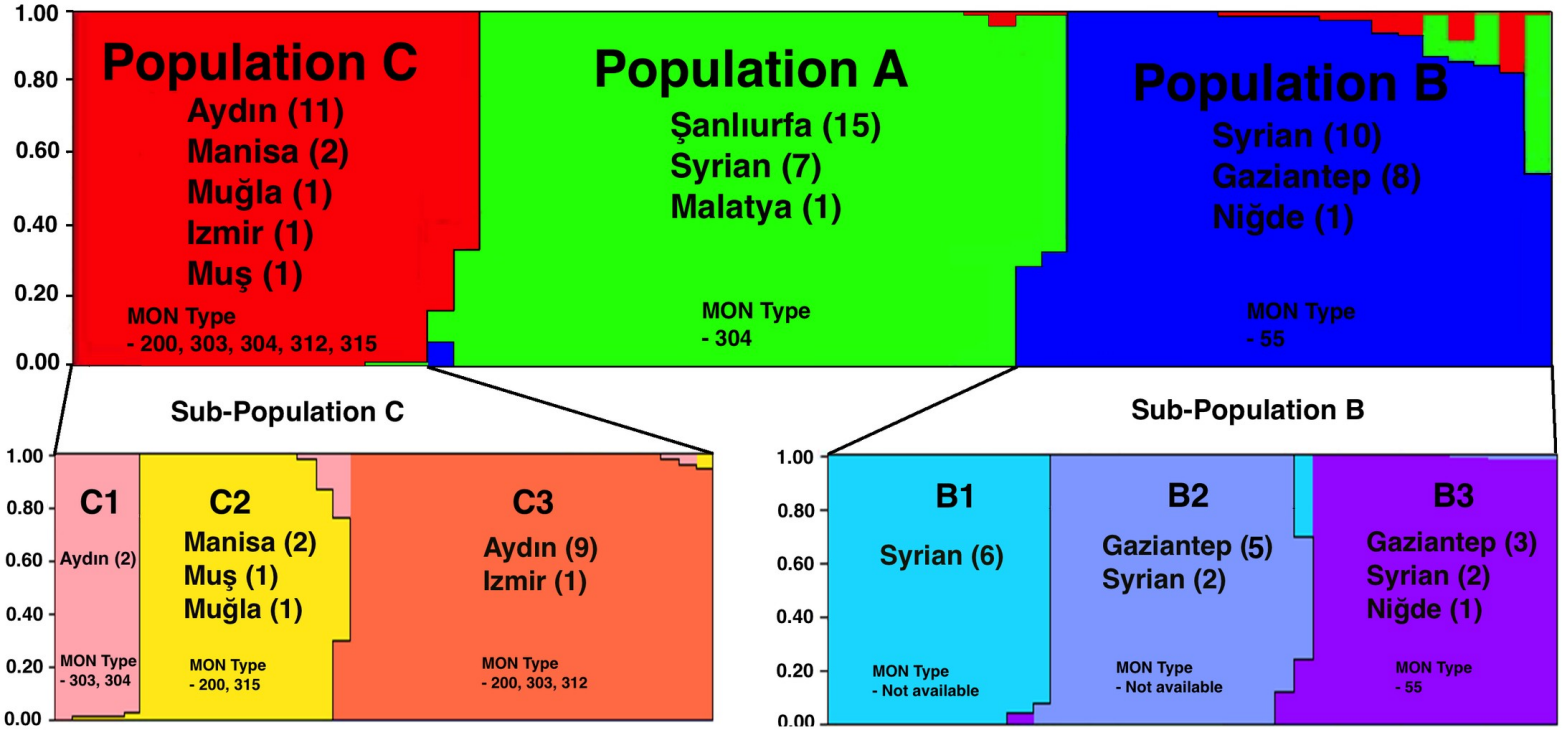
STRUCTURE analysis of the *L*. *tropica* samples.

**Fig 2:**
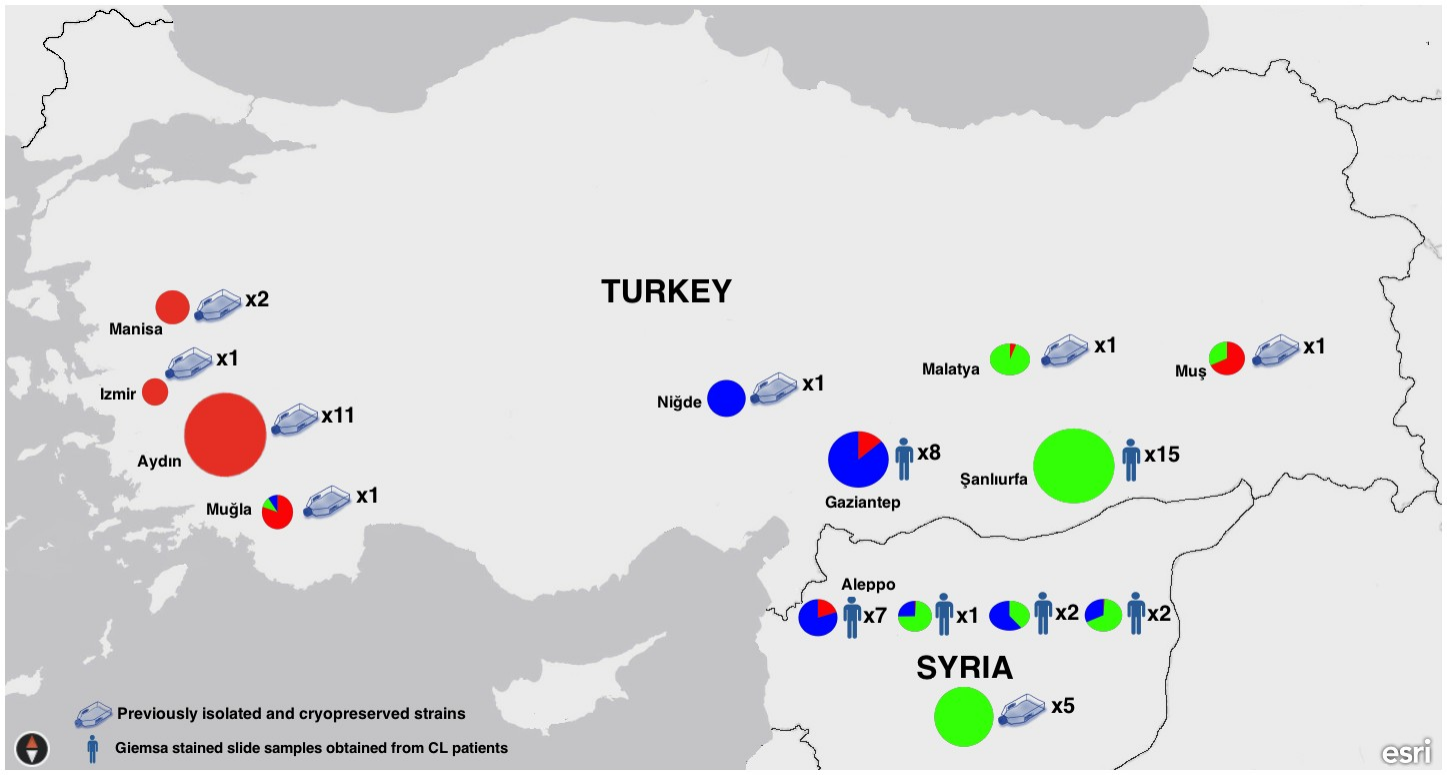
Geographical distribution of MLMT profiles.

Bayesian statistics were applied using STRUCTURE analysis and according to ΔK calculations, three main populations (POP A, POP B and POP C) were identified. Twenty-three isolates were clustered in POP A, while 19 were in POP B and 16 were in POP C. Three subpopulations were identified both for POP B and C, while no subpopulation was identified for POP A (Fig 1). The majority of the populations were correlated with their geographical origins but no clear difference was noted between samples from Şanlıurfa and Syria, where population exchanges occur frequently (Fig 2).

In total, 23 *L*. *tropica* isolates were clustered in main POP A, which were isolated from three different geographical origins (15 Şanlıurfa, seven Syrian and one from Malatya). When POP B is analyzed separately using STRUCTURE, it split into three subpopulations, B1, B2 and B3. POP B comprised of 10 samples from Syrian, eight samples from Gaziantep and one samples from Niğde. All Syrian samples in POP B were clustered as sub-population B1, while all two Syrian and five Gaziantep samples were clustered in sub-population B2. The one sample from Niğde was clustered in sub-population B3 with three Gaziantep and two Syrian samples. Third main POP C comprised all samples originated from Mediterranean except one sample, which clustered in POP C. The sample was isolated from Muş city in eastern Turkey, which geographical origin does not correlate with other samples clustered in POP C. POP C was analyzed separately and three subpopulations were identified. However, the ΔK values suggest weak substructure and the sub-populations did not correlate with their geographical origins.

Descriptive analysis per locus was performed and coefficient of relationship was noted positive for four markers (4GTG, LIST7033, GA1 and GA2) (Tables 1 and 2). The F_IS_ per locus was found positive in 11 out of 12 markers that suggest the large number of homozygous alleles in studied samples. Descriptive analysis per populations (K: 3) was also performed and POP B was found to be the most heterogeneous group by having the highest number of alleles, H_e_ and H_o_ values.

**Table 1:** Descriptive analysis per locus.

Marker	number of strains	repeat array	fragment size array [bp]	A	H_e_	H_o_	F_IS_
GA1	58	GA 11–12	66–68	2	0.159	0	1
GA2	58	GA 5–8	56–62	3	0.443	0	1
GA6	58	GA 8	61	1	0	0	0
GA9n	55	GA 7–9	112–116	3	0.462	0.6	-0.302
LIST7010	46	TA 21–35	176–204	8	0.680	0.174	0.746
LIST7011	56	TA 9–19	174–194	5	0.772	0.018	0.977
LIST7027	56	CA 6–12	169–181	6	0.760	0.518	0.321
LIST7033	58	GT 7–8	178–180	2	0.310	0	1
LIST7039	53	CA 11–28	195–229	9	0.613	0.189	0.694
LIST7040	52	GT 15–24	229–247	3	0.551	0.5	0.093
4GTG	58	GTG 4–6	59–65	3	0.100	0	1
27GTGn	58	GTG 5–9	106–118	4	0.672	0.431	0.360
mean	55.5			4.1	0.460	0.202	0.562

A—number of alleles; H_o_—observed heterozygosity; H_e_—expected heterozygosity; F_IS_—inbreeding coefficient

-1 = outcrossing; 0 = random mating; +1 = inbreeding

**Table 2:** Descriptive analysis per populations.

Marker	number of strains	A	H_e_	H_o_	F_IS_
Pop A	23	1.83	0.254	0.330	-0.308
Pop B	19	3.00	0.466	0.139	0.709
Pop C	16	2.25	0.259	0.074	0.716
mean		2.36	0.326	0.181	0.448

A—number of alleles; H_o_—observed heterozygosity; H_e_—expected heterozygosity; F_IS_—inbreeding coefficient

-1 = outcrossing; 0 = random mating; +1 = inbreeding

One of the *L*. *tropica* strains was viscerotropic while the rest were dermotropic. The MLMT repeat motif and Bayesian clustering method revealed that there are no significant differences between the viscerotropic isolate and the dermotropic isolates. The majority of the amplified markers (11/12) of the viscerotropic isolate were found to be highly similar to those in dermotropic isolates having the same geographical origins. The viscerotropic isolate was clustered in same subpopulation (Sub-Pop C) with those dermatropic isolates.

The isoenzyme analysis (MLEE) was previously performed to 33 out of 38 autochthonous strains and six different profiles (MON-55, MON-200, MON-303, MON-304, MON-312 and MON-315) were identified. POP A contained 16 strains belonging to MON-304 and seven Giemsa-stained smears for which MON typing was not available. POP B contains 18 smears with no MON profile and one sample belonging to MON-55. Five different MON profiles (MON-200, MON-303, MON-304, MON-312 and MON-315) were clustered in population C.

### Combined analysis of new and old Turkish *L*. *tropica* MLMT profiles

**Fig 3:**
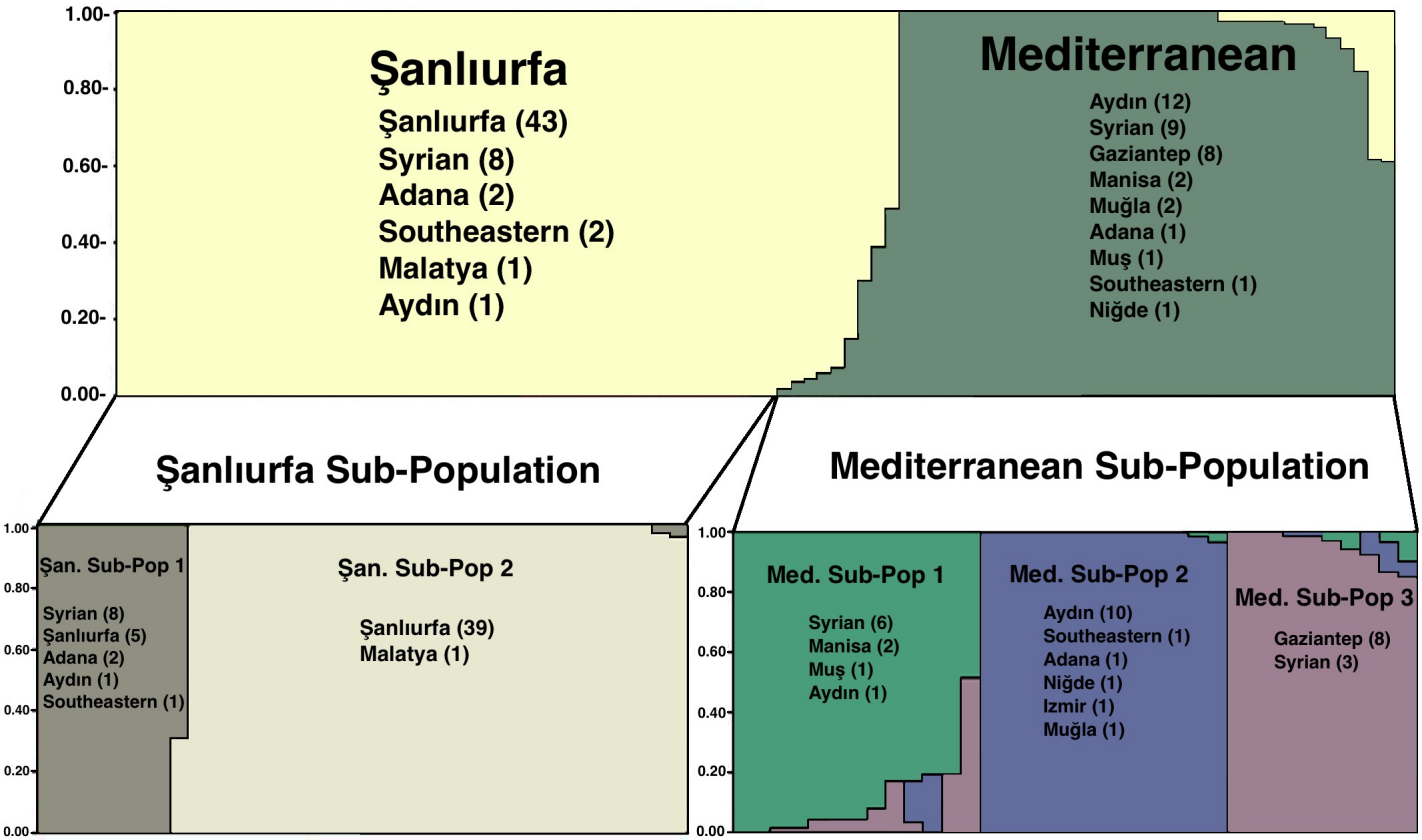
STRUCTURE analysis of the Turkish *L*. *tropica* samples (including previous *L*. *tropica* data).

MLMT data from the previously studied 35 *L*. *tropica* samples [16] were combined with the 58 samples in the present study and all 93 samples reanalyzed using STRUCTURE software and ΔK (K:2) was calculated. Two main populations were observed and they were named as Şanlıurfa and Mediterranean according to the origins of the majority of *L*. *tropica* strains. The populations, Şanlıurfa and Mediterranean were analyzed separately and found to form two and three subpopulations, respectively (Fig 3).

Descriptive analysis per locus was performed and coefficient of relationship for three markers (GA1, LIST7033 and 4GTG) was found higher among studied markers. According to descriptive analysis per populations, 57 isolates were clustered in Şanlıurfa population and 36 were in Mediterranean population (Table 3).

**Table 3:** Descriptive analysis per locus (including previous *L*. *tropica* data).

Marker	number of strains	repeat array	fragment size array [bp]	A	H_e_	H_o_	F_IS_
GA1	93	GA 11–12	66–68	2	0.102	0.000	1.000
GA2	93	GA 5–8	56–62	3	0.317	0.011	0.966
GA6	92	GA 8	61	1	0.000	0.000	0.000
GA9n	89	GA 7–9	112–116	3	0.488	0.742	-0.522
LIST7010	78	TA 21–35	176–204	9	0.603	0.102	0.831
LIST7011	91	TA 9–19	174–194	5	0.680	0.011	0.984
LIST7027	89	CA 6–12	169–181	7	0.737	0.674	0.086
LIST7033	93	GT 7–8	178–180	2	0.210	0.000	1.000
LIST7039	88	CA 11–28	195–229	9	0.452	0.125	0.725
LIST7040	84	GT 15–24	229–247	3	0.575	0.667	-0.160
4GTG	93	GTG 4–6	59–65	3	0.063	0.000	1.000
27GTGn	70	GTG 5–9	106–118	4	0.696	0.500	0.283
mean	87.7			4.25	0.410	0.236	0.427

A—number of alleles; H_o_—observed heterozygosity; H_e_—expected heterozygosity; F_IS_—inbreeding coefficient

-1 = outcrossing; 0 = random mating; +1 = inbreeding

## Discussion

Şanlıurfa province is a well known highly endemic area for CL caused by *L*. *tropica* and Gaziantep is its neighbor city with 150 km in the west. The disease is present for centuries in this geographical area covering the cities located in northern Syria and southern Turkey. The altitude is between 510 and 800 meters in this plain area and there are no natural barriers like mountains [27]. Although *L*. *major* was detected rarely in recent years in Şanlıurfa, the main causative agent is *L*. *tropica* for most of the CL cases similar to Aleppo [3]. The inhabitants of Aleppo and Sanliurfa are relatives who visit each other frequently staying more than one week. In addition, the mass human migration between western, southeastern and Mediterranean regions in Turkey due to temporary summer works explains the wide spread of *L*. *tropica* in these regions. With *Phlebotomus sergenti* as the dominant and proven vector in the area with no animal reservoir detected so far [27], the life cycle of *L*. *tropica* in the southeastern region of Turkey is considered anthroponotic. Similar findings were also reported from Syria [8]. For these reasons, most of our *L*. *tropica* strains and Giemsa-stained smears were obtained from southeastern region and analyzed in microsatellite level together with the strains isolated in other endemic areas of Turkey for better understanding their epidemiological origin and population structure. Giemsa stained smears obtained from Syrian patients showed similarities in marker level between Gaziantep and Şanlıurfa samples, while another five Syrian isolates were genetically identical to Şanlıurfa strains. The microsatellite profiles detected in this study are the reflection of real life related to mass human movements in the area as explained above.

STRUCTURE analysis and Phylogenetic network clustering identified three main populations among the new isolates. The majority of the populations were congruent with their geographical origins. The oldest Şanlıurfa strain (EP-40) used in the present study was isolated in 1999 after the outbreak in this city, and microsatellite typing was performed for the first time. The MLMT data was compared with a more recent strain isolated in 2007 from the same area (EP-142) and no difference was found.

One isolate (EP-171) was viscerotropic and all markers are successfully amplified in the present study. No significant differences were found between other dermatrophic *L*. *tropica* isolates, which clustered in same population. One out of twelve markers was found to be heterozygous in this viscerotropic strain. This difference is statistically insignificant as suggested by STRUCTURE analysis. In order to evaluate the effects of clinical outcomes on microsatellite markers, further studies should be performed using more viscerotropic *L*. *tropica* strains.

Totally, 20 Giemsa-stained smears were included in the present study and twelve of them were obtained from Syrian patients with CL. Unfortunately, no detailed information was available regarding their route of travel and whether the infection was acquired in Turkey or Aleppo, Syria. As seen in (S1 Table) and suggested by STRUCTURE (Fig 3) analysis, Syrian smear samples clustered in two different populations and three subpopulations. Even though all Syrian samples and strains were reported to originate from Aleppo, Syria, the MLMT profile of these samples was not identical. The possible reason for this kind of minor changes in microsatellite markers may be the immune status and capability of the patients. Additionally, it can be due to the clonal isolation of the parasite by culturing as previously reported [28].

Our findings showed that the usage of smear samples have some advantages as follows; MLMT can be carried out more rapid without the need for culturing parasite and populations can be analyzed in more detail, which is also important in the meaning of epidemiology. In the present study, two of smear samples grouped in POP A together with 21 isolates from different areas while other 18 smear samples grouped in POP B together with only one isolate. There are two subpopulations consisting of only smear samples originated from POP B. We believe that the smear and strains obtained from same patients needs to be worked together ideally.

In the present study, all of the cryopreserved isolates were previously typed by MLEE [5]. Three main populations were identified according to their zymodeme profiles, and the MON profiles were found to be partially congruent with Bayesian clustering method. Except one isolate, all MON-304 isolates clustered in POP-A, which are mostly Şanlıurfa isolates. Only one isolate MON-55 clustered in POP-B with other smear samples. In terms of MON profiles, POP-C is quite heterogeneous that contains five different groups. Microsatellite analysis of different MON profiles was studied in a previous study, and *L*. *infantum* MON-1 and non MON-1 group were successfully identified and found to be congruent with MLMT data [29]. However, another study conducted using Algerian strains of *L*. *infantum* reported no association between MON profiles and MLMT profiles [30]. Our results also support the idea that MLMT is the best candidate to be accepted as gold standard by having further discriminatory power [31].

Majority of the studied markers of the Syrian *L*. *tropica* samples were found similar to strains isolated from Şanlıurfa city. While Şanlıurfa has the longest border with Syria, and many people cross the border into Turkey in this region, only one out of 12 (0.8%) Syrian isolates was found identical to the Şanlıurfa strains. Giemsa-stained smears studied from another border city with Syria, Gaziantep, showed that the microsatellite repeat numbers for the studied markers were highly similar to Şanlıurfa and the Syrian samples, but not identical. Slight differences in marker level were observed between the samples from Gaziantep, Syrian and Şanlıurfa, which was also supported by Bayesian clustering analysis (S1 Table). The marker LIST7010 is noted to be highly variable among the studied Syrian samples. Interestingly, this marker was found heterozygote in all studied Syrian strains but homozygous in those smear samples obtained from Syrian CL patients.

Analysis of all the Turkish *L*. *tropica* MLMT data available (39 samples), by STRUCTURE demonstrated the presence of two main populations, Şanlıurfa and Mediterranean. Each population further divided into two and three sub-populations, respectively. The Şanlıurfa population mainly consisted of isolates from southeastern Turkey, whereas the Mediterranean population was quite complex. Samples obtained from Syrian patients were clustered in Şanlıurfa-A sub-population, while sub-population Şanlıurfa-B comprised of samples only isolated from Şanlıurfa (Table 4). A previous MLMT study analyzed *L*. *tropica* strains from different regions revealed that two main parasite populations exist in Turkey. Isolates from Şanlıurfa created a unique subpopulation and all Turkish isolates were clustered in a different population, which differs from Moroccan, but correlates with previous isolates from Israel and Palestine [28]. The MLMT data of the isolates clustered in Şanlıurfa population correlate with findings of previous study and noted to be the major *L*. *tropica* population of Turkey [28]. One *L*. *tropica* strain (TRO-35) isolated from a patient in the most western part (Aydın city) of Turkey clustered in Şanlıurfa population and it shows high similarities in the marker level to those strains isolated from eastern/southeastern part of Turkey. These kinds of discrepancies were also reported in previous studies and might have arisen from being infected with the parasite while visiting the other endemic regions for leishmaniasis [18].

**Table 4:** Descriptive analysis per populations and subpopulations.

**Population**	**number of strains**	**A**	**H**_**e**_	**H**_**o**_	**F**_**IS**_
Mediterranean	36	4.00	0.444	0.099	0.779
Şanlıurfa	57	2.42	0.238	0.328	-0.384
mean		3.21	0.341	0.213	0.375
**Sub-population**	**number of strains**	**A**	**H**_**e**_	**H**_**o**_	**F**_**IS**_
Med. Sub-Pop1	13	1.58	0.156	0.065	0.561
Med. Sub-Pop12	13	3.08	0.389	0.096	0.764
Med. Sub-Pop13	10	2.25	0.367	0.131	0.656
Şan. Sub-Pop11	20	2.42	0.323	0.374	-0.163
Şan. Sub-Pop12	37	1.33	0.169	0.303	-0.815
mean		2.13	0.281	0.194	0.305

A—number of alleles; H_o_—observed heterozygosity; H_e_—expected heterozygosity; F_IS_—inbreeding coefficient

-1 = outcrossing; 0 = random mating; +1 = inbreeding

In conclusion; in comparison to previous studies, our findings suggest that, the epidemiology of *L*. *tropica* is much more complicated. A new population, which comprised of Syrian *L*. *tropica* samples, was reported for the first time in Turkey and data available here will provide epidemiological knowledge to further studies. This study highlights the future role of *L*. *tropica* spread across Europe through the current wave of migration of infected people.

## Supporting information

S1 Table Detailed list of the isolates, Giemsa-stained smears and MLMT data of *L*. *tropica* samples used in the present study. (XLSX)

S2 Table The MLMT fragment sizes obtained in the present study. (XLSX)

S1 Fig The Neighbour-joining tree showing 58 *L*. *tropica* samples (Unrooted Neighbour-joining tree was generated using Chord Distance model and all populations/subpopulations are labelled in different colours). (TIF)

S2 Fig The Neighbour-joining tree showing 93 *L*. *tropica* samples (Unrooted Neighbour-joining tree was generated using Chord Distance model and all populations/subpopulations are labelled in different colours). (TIF)
