# Supplementary figures and images for: Epidemiological analysis of Leishmania tropica strains and giemsa-stained smears from Syrian and Turkish leishmaniasis patients using multilocus microsatellite typing (MLMT)
Source: PLoS Negl Trop Dis. 2017 Apr 12;11(4):e0005538. doi: 10.1371/journal.pntd.0005538 (PMC5402985; doi:10.1371/journal.pntd.0005538)

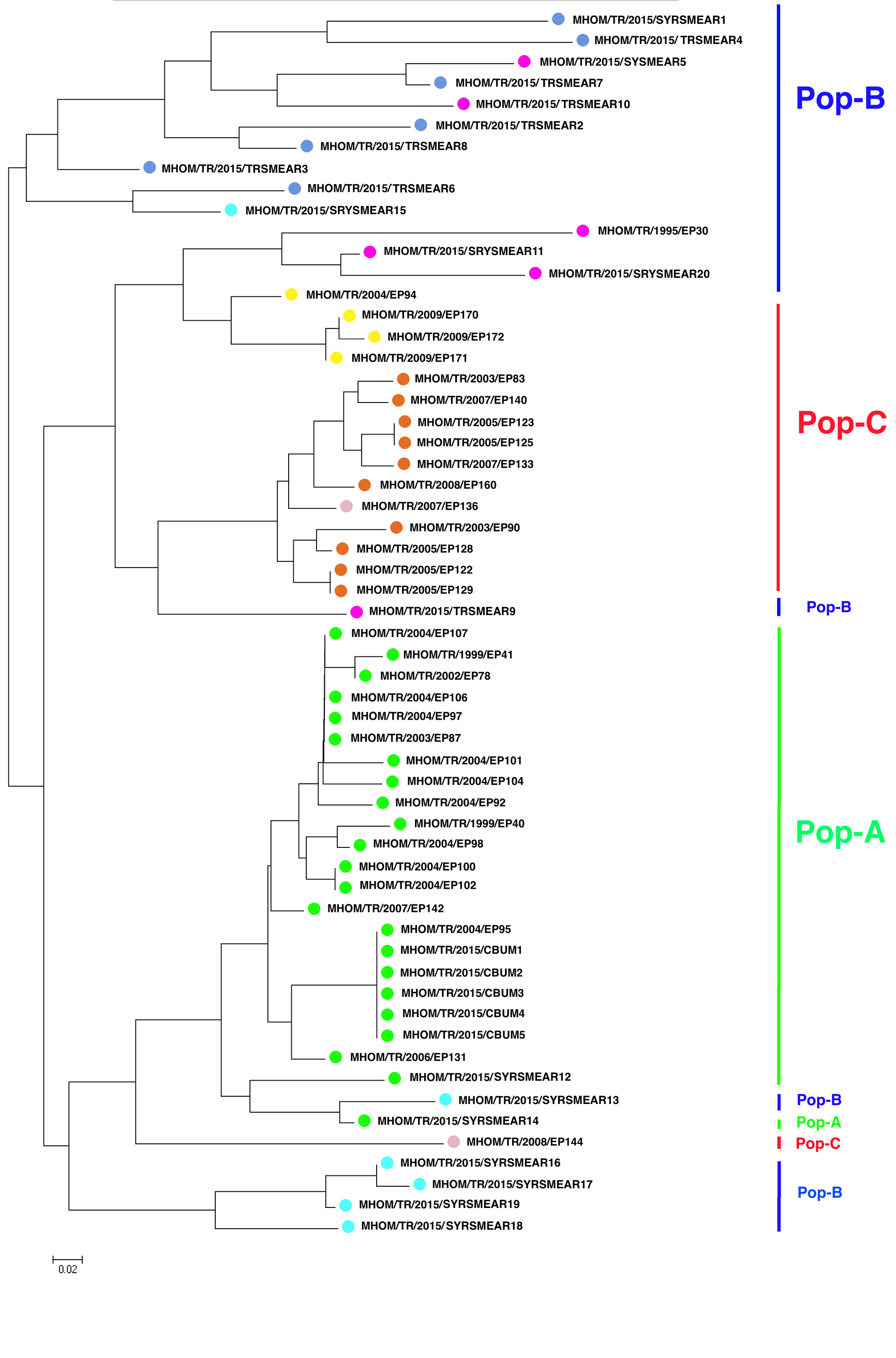

Supplement: S1 Fig — (TIF) [file pntd.0005538.s003.tif]

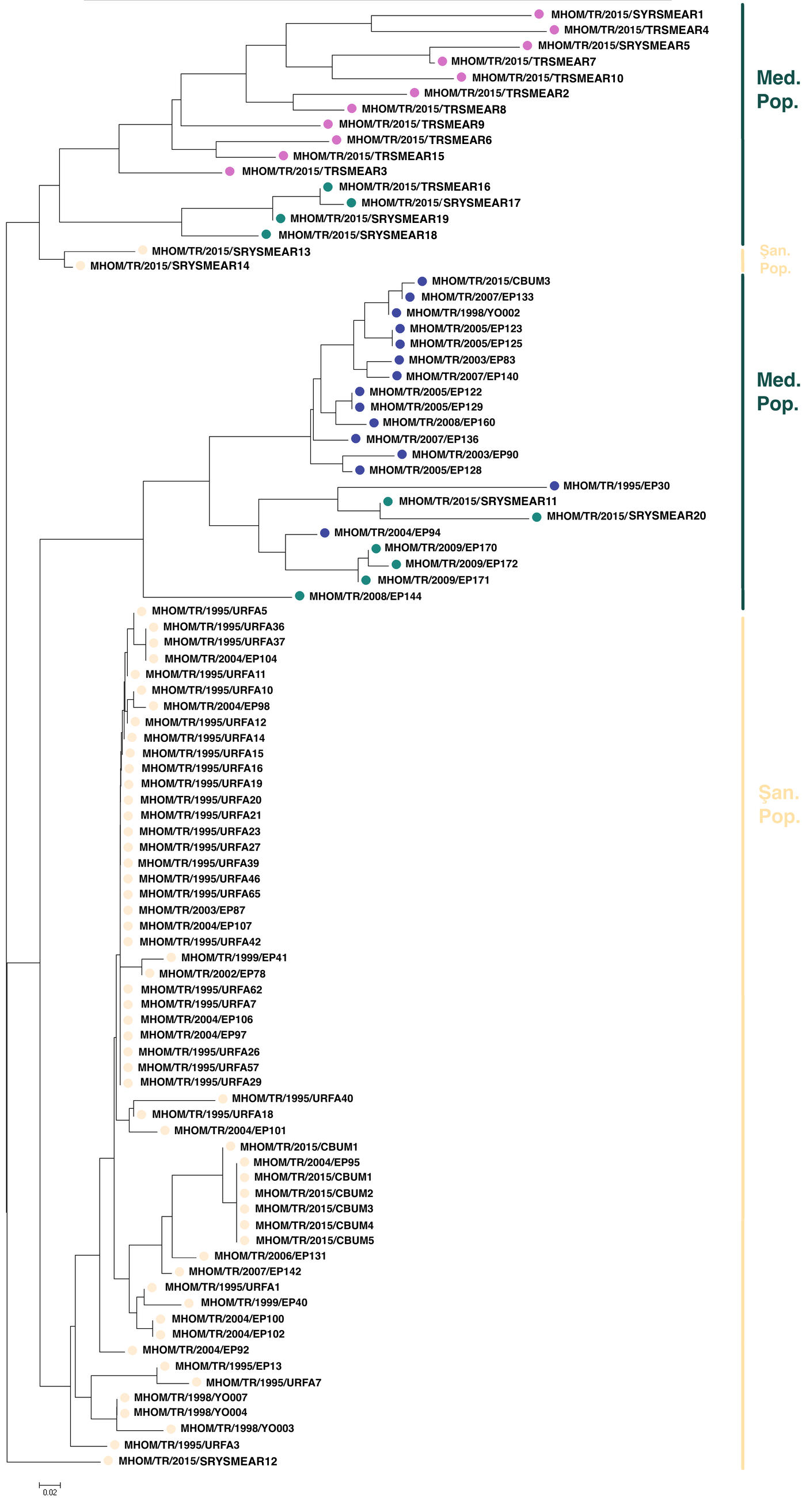

Supplement: S2 Fig — (TIF) [file pntd.0005538.s004.tif]
